# Evaluation of Homocysteine Level as a Risk Factor among Patients with Ischemic Stroke and Its Subtypes

**Published:** 2013-09

**Authors:** Nahid Ashjazadeh, Morteza Fathi, Abdohamid Shariat

**Affiliations:** Shiraz Neurosciences Research Center, Department of Neurology, Chamran Hospital, Shiraz University of Medical Sciences, Shiraz, Iran

**Keywords:** Homocysteine, Risk factors, Vascular disease

## Abstract

**Background:** Epidemiological research has shown that increased total homocysteine (tHcy) levels are associated with an increased risk of thromboembolic disease; however, controversy still exists over which subtype of stroke is allied to hyperhomocysteinemia. This study aimed to investigate whether elevated tHcy is an independent risk factor for ischemic stroke and to compare tHcy levels in patients with ischemic stroke subtypes.

**Methods: **We performed a case-control study, in which 171 ischemic stroke patients aged over 16 years and 86 age and sex-matched controls were eligible to participate and were enrolled from January 2009 to January 2010. The patients’ demographic data, traditional stroke risk factors, and the results of fasting tHcy, vitamin B12, and folate of serum were collected in the first 5 days after ischemic stroke. Stroke subtypes were classified according to the Trial of Org 10172 in Acute Stroke Treatment (TOAST) criteria. SPSS software (version 13) was used for the statistical analysis of the data, and a P value smaller than 0.05 was considered statistically significant.

**Results:** The mean fasting Hcy levels was significantly higher in the cases (16.2 μmol/L, 95% CI: 14.8 to 17.5) than in the controls (13.5 μmol/L, 95% CI: 12.4 to 14.6) (P=0.013). The mean Hcy levels was elevated significantly in those with cardioembolic strokes compared with the controls (17.7 μmol/L, 95% CI: 14.8 to 20.5; P=0.010). The plasma Hcy level was associated with an adjusted odds ratio of 2.17 (95% CI: 1.24 to 3.79; P=0.004) for Hcy above 15 μmol/L concentration for all types of stroke.

**Conclusion: **Our data showed that elevated serum Hcy is an independent risk factor for ischemic stroke and it has a strong association with cardioembolic subtype.

## Introduction

Stroke is a heterogeneous condition and its subtypes have different pathophysiological mechanisms and etiologies. Despite a gradual decline in overall stroke death rates in many industrialized countries, stroke remains a leading cause of death and disability in the world.^1^

Ischemic stroke can be caused by large artery atherosclerotic disease, small vessel or penetrating artery disease (lacunes), cardiogenic or artery-to-artery embolism, nonatherosclerotic vasculopathies, hypercoagulable disorders, or infarcts of undetermined causes. Ischemic strokes account for approximately 80% to 88% of all strokes. The most recognized mechanistic classification is the Trial of Org 10172 in Acute Stroke Treatment (TOAST) classification.^2^

Homocysteine (Hcy) is a four-carbon amino acid with a free thiol group, which is formed by demethylation of methionine, an essential amino acid derived from diet. Normal total Hcy (tHcy) concentrations range from 5-15 µmol/L in the fasting state. Hyperhomocysteinemia (HHcy) has been classified into moderate (plasma tHcy concentrations of 15-30 µmol/L), intermediate (plasma tHcy concentrations of 31-100 µmol/L), and severe (plasma tHcy concentrations 100 µmol/L).^3^ Both acquired and genetic factors can have an impact on plasma tHcy. Male gender, aging, smoking, impaired renal function, and some medications such as Corticosteroids and Cyclosporine are some examples of the acquired causes and classic homocytinuria and C677T homozygote mutation of 5,10-methylenetetrahydrofolate reductase (MTHFR) are the main genetic ones.^3^^-^^6^ Vitamin B12, vitamin B6, and folate, all of which have dietary origins, are three main cofactors in Hcy metabolism. Deficiencies in these supplements are more prevalent in the most developing countries and may account for many cases of moderate hyperhomocysteinemia and increased risk of stroke.^7^ Hao et al.^8^ conducted a study in 2,471 Chinese men and women and showed that decreased plasma levels of folate, vitamin B12, and vitamin B6 as well as male gender and living in urban areas were significantly related to hyperhomocysteinemia. 

Several studies have postulated that elevated tHcy is a strong and independent risk factor for vascular diseases including ischemic cerebral stroke.^1^^,^^3^^,^^9^^-^^16^ Tan et al.^13^ studied 109 young adult Asians (Chinese, Indians, and Malays) with ischemic stroke and found a strong relationship between increased Hcy and ischemic stroke (OR=5.17, 95% CI: 1.96 to 13.63; P=0.001). Other studies have reported the same results in Turkish and Malay populations with ischemic stroke.^17^^,^^18^ Furthermore, Biswas et al.^19^ conducted a study in 120 Indian patients with acute ischemic stroke and showed that there was a significant relationship between HHcy and ischemic stroke (P=0.001). They also found decreased serum concentrations of vitamin B12 and folate in a significant number of their patients and the role of MTHFR 677 C T polymorphisms in hyperhomocysteinemia in some of their patients.^19^

Oxidative damage to the vascular endothelium and the proliferation of the vascular smooth muscle create a prothrombotic condition, which contributes to the development of premature atherosclerosis.^12^^,^^15^^,^^20^ Moreover, HHcy has been found as a potential risk factor for cardiovascular disease and vascular dementia.^21^^-^^24^

Some studies have shown that even mildly increased plasma tHcy can also be a significant risk factor for stroke, more specifically ischemic stroke.^3^ The aim of this study was to evaluate HHcy as a risk factor for ischemic stroke and its relationship to specific subgroups of stroke in an Iranian population.

## Patients and Methods

*Patients and Controls*

From January 2009 to January 2010, this case-control study was conducted in 171 patients aged over 16 years within 5 days of their first ischemic stroke in Nemazee Hospital, affiliated to Shiraz University of Medical Sciences. Each case was evaluated by brain computed tomography (CT) within 24 hours of admission and by duplex ultrasound of extracranial vessels and echocardiography (transthoracic or transesophageal) within the next 3 post-stroke days. Brain magnetic resonance imaging (MRI) with MR angiography was performed in some cases. Controls included 86 age and sex-matched persons without ischemic stroke, who visited our Pathobiology Laboratory for blood sampling. Baseline demographic data (age and sex) and conventional cardiovascular risk factors, including diabetes mellitus (DM), hypertension (HTN), hyperlipidemia (HLP), smoking, and previous coronary diseases, were recorded for the patients and controls. All the patients and controls gave their written informed consent, and the Medical Research Ethics Committee of Shiraz University of Medical Sciences approved the study (approval number: 2817). 

*Sample Collection*

Fasting blood samples were obtained from all the patients within 5 days of ischemic stroke and were immediately chilled on ice. Serum samples were collected within 30 minutes and were thereafter stored at -80ºC. The axis homocysteine enzyme immunoassay (EIA) (Axis-Shield Diagnostics Ltd., United Kingdom) was used for the quantitative analysis of total L-homocysteine in serum. Vitamin B12 and folate were measured using the SimulTRAC-SNB Radioassay Kit (DRG Instruments GmbH, Germany).

*Exclusion Criteria for Cases and Controls*

The exclusion criteria were concomitant history of previous ischemic strokes, cerebral venous infarcts, ischemic heart disease, peripheral vascular disease, hypothyroidism, epilepsy, renal impairment, pregnancy, postpartum state, consumption of oral contraceptives or drugs that might affect serum vitamin B12, folate, and Hcy levels, brain mass or any malignancy, history of migraine, and vitamin B12 and folate deficiencies.

*Stroke Subtypes*

According to the TOAST criteria, stroke subtypes were classified into large artery, cardioembolic, small artery/lacunar strokes, and strokes of other undetermined etiologies.^2,25^ All the patients were subtyped using a modified TOAST criterion.^12^ Patients with incomplete data because of early death or other causes were gathered in the last group. 

*Statistical Analysis*

All the analyses were done using SPSS (version 13) software (SPSS, Inc.). Student’s *t* test was used for the quantitative variables. Chi-square test was used to analyze the qualitative findings. Odds ratios (OR) and 95% confidence intervals were calculated and a P value smaller than 0.05 was considered statistically significant. Age, sex, DM, and smoking were matched in both cases and controls (table 1). HTN and HLP were controlled by stratifying two levels. Binary logistic regression analysis was used to calculate Hcy in the stroke subgroups and controls. Additionally, the analysis of variance (ANOVA) was employed to compare the mean values of B12, folate, and Hcy.

**Table 1:** Demographic data, risk factors, and serum Hcy, vitamin B12, and folate levels in the cases and controls

	**Cases (n=171)**	**Controls (n=86)**	**P value**
Mean age, yr (±SD)	67.9 (13.3)	68.7 (8.4)	0.607
Gender, n (%)			
Male	74 (43.3)	35 (40.7)	0.789
Female	97 (56.7)	51 (59.3)	
Diabetes mellitus, n (%)			
No	125 (73.1)	62 (72.1)	0.883
Yes	46 (26.9)	24 (27.9)	
Hypertension, n (%)			
No	67 (39.2)	61 (70.9)	<0.001
Yes	104 (60.8)	25 (29.1)	
Hyperlipidemia, n (%)			
No	124 (72.5)	48 (55.8)	0.011
Yes	47 (27.5)	38 (54.2)	
Smoking, n (%)			
No	150 (87.7)	74 (86.0)	0.697
Yes	21 (22.3)	12 (14.0)	
Homocysteine (μmol/L)			
Median	13.6	11.7	0.013
(Range)	(3.8-54.1)	(7.0-31.9)	
Mean	16.2	13.5	
(95% CI)	(14.8-17.5)	(12.4-14.6)	
Vitamin B12 (pmol/L)			
Median	242.9	328.7	0.083
(Range)	(62.0-1400)	(63.0-1100)	
Mean	327.3	386.1	
(95% CI)	(286.0-368.5)	(333.4-438.8)	
Folate (nmol/L)			
Median	6.00	6.35	0.908
(Range)	(2.7-18.0)	(2.90-15.30)	
Mean	6.52	6.56	
(95% CI )	(6.12-6.92)	(6.06-7.05)	

## Results

One hundred seventy-one consecutive patients and 86 age and sex-matched controls from the same geographic area were selected. Table 1 shows the baseline demographic values, conventional vascular risk factors, fasting serum Hcy, vitamin B12, and folate levels in the cases and controls. Table 2 illustrates fasting serum Hcy, vitamin B12, and folate levels in the stroke subtypes and controls.

**Table 2:** Fasting serum homocysteine, vitamin B12, and folate levels in stroke subtypes and controls

**Stroke subtype**	**Large-vessel** **(n=24)**	P*	**Cardioembolic** **(n=56)**	P*	**Small-vessel/Lacunar** **(n=33)**	P*	**Undetermined causes** **(n=32)**	P*	**Other causes** **(n=26)**	P*	**Controls** **(n=86)**
Mean Hcy level (μmol/L) (95% CI)	13.7 (10.6-16.8)	1.000	17.7 (14.8-20.5)	0.010	14.2 (11.7-16.6)	0.988	16.4 (13.3-19.5)	0.081	17.4 (13.5-21.3)	0.029	13.5 (12.4-14.6)
Mean B12 level (pmol/L) (95% CI)	225.7 (163.8-287.6)	0.576	387.9 (297.4-478.3)	1.000	332.3 (239.3-425.3)	0.753	289.5 (207.3-371.7)	0.355	330.9 (227.9-433.9)	0.569	386.1 (333.4-438.8)
Mean folate level (nmol/L) (95% CI)	5.7 (4.9-6.5)	0.390	7.0 (6.2-7.8)	0.735	6.4 (5.6-7.2)	0.993	6.2 (5.3-7.0)	0.874	6.9 (5.7-8.0)	0.733	6.56 (6.1-7.1)

*Dunette-Test, comparing each group with the controls separately

In this study, our findings showed that mean levels of fasting serum Hcy were significantly higher in the cases than in the controls (16.2 μmol/L vs. 13.5 μmol/L; P=0.013). The mean Hcy level was significantly higher in the cardioembolic group than in the controls after adjustment for HTN and HLP (17.7 μmol/L vs. 13.5 μmol/L; P=0.008). No other stroke subtypes showed significantly different Hcy levels after adjustment compared with the controls. There was a significant difference in vitamin B12 level between the large vessel subgroup and the controls before adjustment for HTN and HLP (P=0.033), but the difference was not significant after adjustment. Also, the difference in folate level between the cases and controls was not statistically significant (6.52 nmol/L vs. 6.56 nmol/L; P=0.908). Our study showed that fasting Hcy had a strong, graded, and independent relationship with the risk of ischemic stroke. The odds ratio of 2.17 (95% CI: 1.24 to 3.79; P=0.004) for Hcy above 15 μmol/L concentration for all types of stroke was achieved. Fasting Hcy was also a strong risk factor for the cardioembolic subtype (OR=2.8, 95% CI:1.4 to 5.6; P=0.05) for Hcy above 15 μmol/L in our patients, but not for the large vessel or lacunar or the other undetermined categories.

## Discussion

Over the last decade, convincing evidence has been gathered on the relation between moderate elevation of plasma tHcy and ischemic stroke. Several studies have reported that HHcy is associated with two to threefold increased risk of ischemic stroke.^3^^,^^13^^,^^15^^,^^26^ In 1995, Boushey et al.^26^ reported the results of the first meta-analysis of 27 observational studies on Hcy and atherosclerotic vascular disease, of which 11 studies addressed the association between Hcy and risk of stroke. Nine case-control studies provided support for the hypothesis that Hcy is an independent risk factor for stroke, while 2 prospective studies reported negative results. Similar to our findings, the odds ratio of this meta-analysis for cerebrovascular disease in patients with elevated Hcy levels was 2.5 (95% CI, 2.0 to 3.0). In 6 studies with fasting blood samples, the odds ratio for a 5 μmol/L increment in Hcy showed that there was an approximately twofold increase in risk (OR=1.9; 95% CI, 1.6 to 2.3).^11^ Similar to our findings, several Asian studies have shown the independent role of HHcy in increasing the risk of ischemic strokes.^13^^,^^17^^-^^19^^,^^27^ However, some of these studies have had the confounding effects of nutritional deficiencies (such as vitamin B12, vitamin B6, and folate).^13^^,^^17^^,^^19^ Omrani et al.^28^ conducted a study in 93 Iranian patients with acute ischemic stroke and concluded that HHcy was a risk factor for ischemic stroke. They did not study the relationship between HHcy and ischemic stroke subtypes, but showed that there was a significant relationship between HHcy and smoking in their patients group.

Studies which have evaluated the relationship between Hcy levels and stroke subtypes have shown different results. A Swedish study in 57 stroke patients with HHcy reported significantly higher tHcy in all stroke subtypes.^11^ Eikelboom et al.^29^ reported that tHcy was significantly greater in large artery and small vessel stroke compared with cardioembolic and controls. Tan et al.^13^ showed that increased tHcy was associated with a higher risk of large artery stroke.^13^ Two other studies in a Turkish population demonstrated that HHcy had a significant role in lacunar and large vessel atherothrombotic, increased intimal media thickness of extracranial carotid arteries, and severe carotid stenosis.^18^^,^^27^ Other studies have shown a relation between increased tHcy and lacunar stroke and carotid stenosis.^12^^,^^30^^,^^31^

Our findings on the relationship between HHcy and cardioembolic subgroup may be explained by higher prevalence of cardiac disease in our country or the fact that our center is a referral center and most uncomplicated patients that have fewer vascular risk factors are not referred to this center. These findings may support the hypothesis that HHcy has different mechanisms of pathogenicity, which may show the influence of other undiagnosed genetic and environmental factors acting as confounders.

Several factors contribute to increased plasma Hcy levels. Individuals with pre-existing atherosclerosis have higher Hcy levels than those without pre-existing atherosclerosis. It seems that there is an association between economic prosperity and the risk of stroke. Higher prevalence of HHcy in many developing countries could indicate the role of inadequate intake of vitamins and antioxidants in the multi-factorial causes of stroke.^3^^,^^7^ The effect of genetic factors on hyperhomocysteinemia is also important. In fact, these factors may confound the results of epidemiological studies and may render the results statistically unstable.^32^

This study has some important limitations. First, intracranial atherosclerosis can give rise to lacunar infarcts indistinguishable from lacunes and may result in small vessel/lacunar misclassification. Furthermore, small cardioembolic emboli can cause lacunar syndromes, acting as a confounding factor in the analysis of the relation between HHcy and stroke subtypes. We also could not omit HHcy as an acute-phase reactant and possible genetic propensity of our patients to HHcy.

In our study, we tried to match all the previously known traditional risk factors of cerebrovascular disease in the selection of controls. However, it was achieved only for age, sex, DM, and smoking and we resolved the confounding actions of HTN and HLP with statistical methods. 

It is deserving of note that no randomized trial has so far shown that lowering tHcy reduces the prevalence of cerebral ischemic events.^11^ Boushey et al.^26^ demonstrated that the administration of folate, vitamin B12, and vitamin B6 decreased Hcy. Furthermore, Biswas et al.^19^ reported that taking 5 mg folate decreased Hcy significantly. However, large randomized trials are needed to determine whether decreased tHcy levels by multivitamin therapy can reduce the risk of cardiovascular disease. If a combination of vitamins is found to be effective, this safe, inexpensive, easily administered therapy will probably be widely used throughout the world and have a major effect on public health.

## Conclusion

This study showed that an elevated Hcy level was an independent risk factor for ischemic stroke in patients who live in the Iranian province of Fars. In addition, there was a significant relationship between increased Hcy levels and the risk of cardioembolic strokes. 
